# Auxin, Strigolactones, and Nitric Oxide Coordinate Lateral Root Development in Rice During Early Interaction With *Rhizophagus irregularis*

**DOI:** 10.1111/ppl.71099

**Published:** 2026-09-08

**Authors:** Giulia Raffaele, Marilena Ronzan, Emanuela Del Dottore, Jian You Wang, Paolo Bianchini, Alberto Diaspro, Carlo Filippeschi, Salim Al‐Babili, Laura De Gara, Barbara Mazzolai

**Affiliations:** ^1^ Bioinspired Soft Robotics Laboratory Istituto Italiano di Tecnologia Genova Italy; ^2^ Department of Science and Bio‐Technology Università Campus Bio‐Medico di Roma Rome Italy; ^3^ Department of Biology Università degli Studi di Napoli Federico II Napoli Italy; ^4^ The BioActives Lab King Abdullah University of Science and Technology Thuwal Saudi Arabia; ^5^ Agricultural Biotechnology Research Center Academia Sinica Taipei Taiwan; ^6^ Biotechnology Center in Southern Taiwan Academia Sinica Tainan Taiwan; ^7^ Nanoscopy and NIC@IIT Istituto Italiano di Tecnologia Genova Italy; ^8^ DIFILAB, Department of Physics Università di Genova Genova Italy

**Keywords:** auxin, large lateral roots, nitric oxide, *Oryza sativa*, *Rhizophagus irregularis*, strigolactones

## Abstract

Arbuscular mycorrhizal fungi (AMF) form symbiotic associations with plant roots, profoundly shaping root system architecture (RSA) and influencing nutrient acquisition in crops. This modulation begins during the early pre‐symbiotic stage, when plants and fungi interact without physical contact. Root formation is orchestrated by signaling molecules, including hormones such as auxin (IAA) and strigolactones (SLs), as well as reactive nitrogen species such as nitric oxide (NO). However, the mechanisms by which AMF spores modulate these pathways to influence root development in rice remain largely unexplored. Here, we investigated the effects of *Rhizophagus irregularis* spores on root formation in *Oryza sativa* L., focusing on IAA, SLs, and NO modulation. Exposure to both live and autoclaved spores enhanced lateral root formation in adventitious roots, whereas only live spores promoted elongation and secondary branching of large lateral roots (LLRs), a rice‐specific feature. These effects were correlated with increased IAA levels, transcriptomic changes, and decreased SL accumulation, revealing an integrated signaling network controlling LLR development. Histochemical analyses revealed NO accumulation in the root elongation zone and apex, accompanied by the upregulation of the high‐affinity nitrate transporter *OsNRT2.1* in LLRs. Together, our findings reveal a root‐type‐specific involvement of IAA, SLs, and NO in shaping RSA during the pre‐symbiotic stage of AMF interactions. This study provides new insights into early signaling events that mediate host discrimination and regulate root architecture during the pre‐symbiotic phase of AMF establishment.

## 1 Introduction

Root formation and development are dynamic processes that depend on plant genetics but are also shaped by environmental factors and biotic interactions, including symbiotic relationships with arbuscular mycorrhizal fungi (AMF; Gonin et al. 2023; Zhu et al. 2025). Phytohormones and signaling molecules act as mediators, transducing environmental cues into adaptive responses in the root system (Altamura et al. 2023; Jan et al. 2024). AMF establish mutualistic associations with plant roots, enhancing nutrient acquisition and stress tolerance (Bhupenchandra et al. 2025). These interactions often trigger significant changes in root system architecture (RSA), including increases in lateral root (LR) formation and development (Sun et al. 2015; Chiu et al. 2018, 2022), thereby optimising the root soil interface for symbiosis. During the pre‐symbiotic phase, germinating AMF spores release signaling molecules called myc‐factors that initiate root morphological responses, promoting fungal colonization of root tissues and nutrient exchange (Aparicio Chacón et al. 2023). Examples of root plasticity mediators are the phytohormones auxin (IAA), strigolactones (SLs), and the signaling molecule nitric oxide (NO; Lanfranco et al. 2018), whose interaction has been highlighted in lateral and adventitious root formation in sunflower seedlings, suggesting their functional cooperation (Bharti and Bhatla 2015).

IAA plays a direct role in root development during plant‐microbe interactions (Lin et al. 2023). In AMF associations, IAA biosynthesis is induced in host plants, leading to enhanced LR development and root hair elongation (Ludwig‐Müller 2010; Hanlon and Coenen 2011; Liu et al. 2018). More broadly, IAA regulates primary and lateral root formation by promoting cell division and elongation (Nakamura et al. 2023). In rice, IAA specifically drives the formation of large lateral roots (LLRs), a distinct lateral root type that develops from adventitious roots alongside fine lateral roots (FLRs), providing preferred sites for symbiosis (Kawai et al. 2022).

SLs are carotenoid‐derived phytohormones involved in AMF symbiosis (Fiorilli et al. 2019; Mitra et al. 2021). They were initially identified as germination stimulants for parasitic plants such as *Striga* and *Orobanche* (Shi et al. 2026), but have since been recognized for their broader role in plant biology. These roles include the regulation of shoot and root architecture (Aquino et al. 2021; Ito et al. 2022; Chen et al. 2023), the promotion of AMF colonization (Foo et al. 2013; Kobae et al. 2018; Wang, Chen, Braguy, and Al‐Babili 2024), and the enhancement of fungal hyphal branching (Akiyama et al. 2005). In rice root architecture regulation, SLs interact with IAA by regulating primary and adventitious root elongation, lateral root formation, secondary root growth, and AR initiation (Cheng et al. 2013; Lakehal and Bellini 2019). Furthermore, in rice, expression of SL biosynthetic genes was observed in LLRs, but not in FLRs, in response to AMF (Fiorilli et al. 2015).

Recent studies have shed light on the differences between canonical and noncanonical SLs in rice (Wang et al. 2022, 2024; Wang, Balakrishna, Martínez, et al. 2024; Li, Haider, et al. 2024; Li, Chen, et al. 2024). The canonical SLs, 4‐deoxyorobanchol (4DO) and orobanchol (Oro), play key roles in rhizosphere communication, for example by promoting AMF colonization, whereas both canonical and noncanonical SLs function as host‐recognition signals (Yoneyama et al. 2018). Recent findings indicate that SLs can influence NO homeostasis by regulating S‐nitrosoglutathione reductase (GSNOR), an enzyme controlling NO levels, thereby modulating root growth responses (Oláh et al. 2020).

NO is a gaseous free radical, increasingly recognized as a key signaling molecule because of its role in several metabolic and physiological processes, as well as in lateral and adventitious root development and root primordia formation (Piacentini et al. 2020; Altamura et al. 2023). In the context of mycorrhizal symbiosis, NO contributes to both the establishment and regulation of AMF symbiosis, specifically by triggering the common symbiotic signaling pathway (SYM) in the host plant after symbiont perception (Martínez‐Medina, Pescador, Fernández, et al. 2019; Martínez‐Medina, Pescador, Terrón‐Camero, et al. 2019).

Several studies have reported the occurrence of a NO burst both during the early stages of AM symbiosis and during initial root interactions with mutualistic fungal endophytes (Zou et al. 2017; Martínez‐Medina, Pescador, Fernández, et al. 2019; Martínez‐Medina, Pescador, Terrón‐Camero, et al. 2019; Pathak et al. 2024). The specific functions of NO as a signaling molecule in the initial phases of AMF–plant interactions remain unresolved and merit further study. In relation to root development, NO closely interacts with phytohormone pathways, enhancing IAA synthesis and transport, thereby influencing IAA‐induced root formation and modulating SL levels to fine‐tune root developmental processes (Bouzroud et al. 2018; Sanchez‐Corrionero et al. 2023).

Therefore, given the limited knowledge regarding how AMF directly affect root formation and development through internal signaling molecules, this study aimed to investigate the involvement of the phytohormones IAA and SLs, together with NO, during the pre‐symbiotic phase in rice. To address this, we exposed rice roots to both non‐autoclaved and autoclaved AMF spores and combined morphological, hormone quantification, histochemical, and transcriptomic analyses. Our results reveal a root‐type‐specific response to spore exposure, driven by these signaling molecules.

## 2 Materials and Methods

### 2.1 Biological Material and Growth Conditions

Seeds of *Oryza sativa* L. ssp. *Japonica* (cv. Nipponbare) were surface sterilized by using a two‐step procedure. First, seeds were sterilized with a 70% (v/v) ethanol solution for 1 min 30 s and then rinsed three times with Milli‐Q water. This was followed by a second sterilization step using a 40% (v/v) sodium hypochlorite solution for 25 min, after which the seeds were again rinsed three times with Milli‐Q water. Afterwards, the seeds were sown in sterile Phytatray containers (Sigma‐Aldrich). The culture medium consisted of half‐strength Murashige and Skoog (Murashige and Skoog 1962), supplemented with 0.1% sucrose and 0.8% agar, adjusted to a pH 5.6–5.8. The medium was autoclaved at 120°C and 1 atm for 20 min and poured under the laminar‐flow hood. Thirty seeds per treatment were sown on the medium and kept under long‐day conditions (14 h light/10 h dark) at 28°C and 60% humidity for 6 days.

Spores of the mycorrhizal fungus *Rhizophagus irregularis* (MUCL 41833), formerly *Glomus intraradices*, were obtained from the Glomeromycota IN vitro COllection (GINCO) of the Université Catholique de Louvain, Belgium. The inoculum was stored in the dark at 4°C and handled under sterile conditions.

Six days after germination, *Oryza sativa* L. seedlings were transferred to square Petri dishes containing Modified Strullu and Romand (MSR) medium (Declerck et al. 1998), solidified with 5% agar, and either exposed or not exposed to *Rhizophagus irregularis* spores for 7 days. In addition to the control plants (C), the treated plants were inoculated with 100 μl of autoclaved water containing either 100 non‐autoclaved spores (S) or 100 autoclaved spores (AS). The AS were obtained by autoclaving the spore samples at 120°C and 1 atm for 20 min (Figure S1). The S and AS groups were used to determine whether plant responses were specifically induced by metabolically active fungal signals or could be triggered merely by the presence of spores. The number of spores used was chosen based on preliminary experiments performed with 100, 300, and 500 spores. The 100 spores' treatment induced the strongest morphological response in root system architecture (Figure S2).

### 2.2 Morphological Analysis

Morphological analysis was carried out on 30 seedlings either not exposed (C) or exposed to *Rhizophagus irregularis* spores (S and AS) for 7 days. Root systems were harvested and fixed in 70% (v/v) ethanol. Mean fresh weight of roots and shoots, root number, embryonic adventitious roots (ARs) length and the number and density of LLRs, including root primordia (RP), secondary roots, secondary lateral roots and primordia were evaluated using a Nikon SMZ745T stereomicroscope coupled with DC500 camera and analysed with the NIS‐Elements Nikon Software. To visualize endodermal cell lengths in LLRs, samples were cleared with chloral hydrate solution (Goodrich 2002) mounted on microscope slides and observed using a Nikon Eclipse Ti2 microscope.

### 2.3 Phytohormone Detection: IAA and SLs

For IAA and SL quantification, ~30 mg of rice roots and shoots from control (C) and *Rhizophagus irregularis*‐treated plants (S and AS) were harvested, flash‐frozen in liquid nitrogen, and stored at −80°C. Extraction and analysis were performed according to previously established protocols (Ito et al. 2022; Chen et al. 2023). For SLs, lyophilized and ground root tissues were spiked with 2 ng rac‐GR24 and extracted twice with 2 ml ethyl acetate using ultrasonication (15 min), followed by centrifugation (8 min, 1292 g, 4°C). Combined supernatants were dried, redissolved in ethyl acetate and hexane, and purified using a silica SPE column. After elution with ethyl acetate and evaporation, samples were reconstituted in acetonitrile: water (25:75, v/v), filtered (0.22 μm), and subjected to LC–MS/MS analysis. For IAA, freeze‐dried ground tissues were spiked with 10 ng D2‐IAA and extracted twice with methanol (750 μl) using ultrasonication (15 min) and centrifugation (5 min, 14 000 × g, 4°C). Combined supernatants were dried, reconstituted in acetonitrile: water (25:75, v/v), filtered, and analyzed by LC–MS. SLs and IAA were quantified using a UHPLC triple quadrupole mass spectrometer (Thermo Scientific Altis) with a Hypersil GOLD C18 column (150 × 4.6 mm, 3 μm). Mobile phases consisted of water and acetonitrile (both with 0.1% formic acid) at a flow rate of 0.5 ml min^−1^. Gradient elution was 25%–100% B (15 min) for SLs and 15%–100% B (10 min) for IAA. The injection volume was 10 μl, and the column temperature was maintained at 35°C. Detection was performed in positive ion mode using H‐ESI with optimized MS parameters. MRM transitions included 176.2→130 for IAA and 178.2→132 for D2‐IAA, along with established transitions for SL compounds and *rac*‐GR24 (Wang et al. 2026). All analyses were conducted with three independent biological replicates per treatment.

### 2.4 NO Detection and Spore Staining

NO production was determined using the specific fluorescent NO probe 4‐amino‐5‐methylamino‐2′,7′‐difluororescein diacetate (DAF‐FM DA; Sigma‐Aldrich). In addition, treatment with 100 μM 2‐(4‐carboxyphenyl)‐4,4,5,5‐tetramethylimidazoline‐1‐oxyl‐3‐oxide (cPTIO), a well‐known NO scavenger, was used as a negative control.

Briefly, rice roots either not exposed or exposed to S and AS, followed or not followed by a cPTIO treatment, were incubated with 10 μM DAF‐FM DA. After 30 min of incubation at 25°C, roots were washed three times with 20 mM HEPES/NaOH buffer to remove excess probe and immediately observed using a Nikon Eclipse Ti2 widefield fluorescence microscope (Nikon Instruments) equipped with a Nikon Digital Sight F camera.

LED excitation was set at 480 nm, whereas fluorescence emission was collected within the 500–550 nm spectral window using an S Plan Fluor ELWD 20×/0.45 air objective.

To detect NO fluorescence localisation across LLR sections, three‐dimensional and time‐lapse confocal imaging were performed using the Nikon AX/AX R Confocal Microscope System equipped with the NSPARC system (Nikon Instruments), which improves resolution through image‐scanning microscopy (Castello et al. 2019).

Z‐stacks were acquired using a z‐step of 0.21 μm and an Apo LWD 40× WI IS DICN2 objective, with excitation at 445 nm and emission collected between 505 and 545 nm. Relative fluorescence intensity in LLRs was quantified using ImageJ software (version 1.53c; Wayne Rasband, National Institutes of Health).

Autoclaved spores of *R. irregularis* were stained with Nitroblue Tetrazolium (NBT) and acid fuchsin contrast solution (Figure S1). Enzymatic activity in viable spores reduces NBT to formazan, producing a dark blue to black precipitate within spores, whereas autoclaved spores remained colorless, transparent, or faintly yellowish (Cornejo and Aponte 2020), thereby confirming their inactive state. Stained spores were mounted on glass slides and observed using the Nikon Eclipse Ti2 microscope.

### 2.5 Transcriptomic Analysis

For transcriptome sequencing analysis, approximately 30 mg of LLR samples were collected, frozen in liquid nitrogen, and stored at −80°C. RNA extraction, sequencing, and statistical analyses were performed by an external provider (Novogene Co. Ltd.). Analyses were conducted using three independent biological replicates per treatment.

### 2.6 Statistical Analyses

The results presented derive from the average of three independent biological replicates, which showed comparable trends. Data are presented as mean ± standard error (SE). Statistical analyses were performed using one‐way parametric ANOVA followed by Tukey's multiple‐comparisons test with GraphPad Prism software (version 8.0.2; GraphPad Software).

## 3 Results

### 3.1 Spore Exposure Specifically Enhanced Root Biomass and Large Lateral Root Density

**FIGURE 1:**
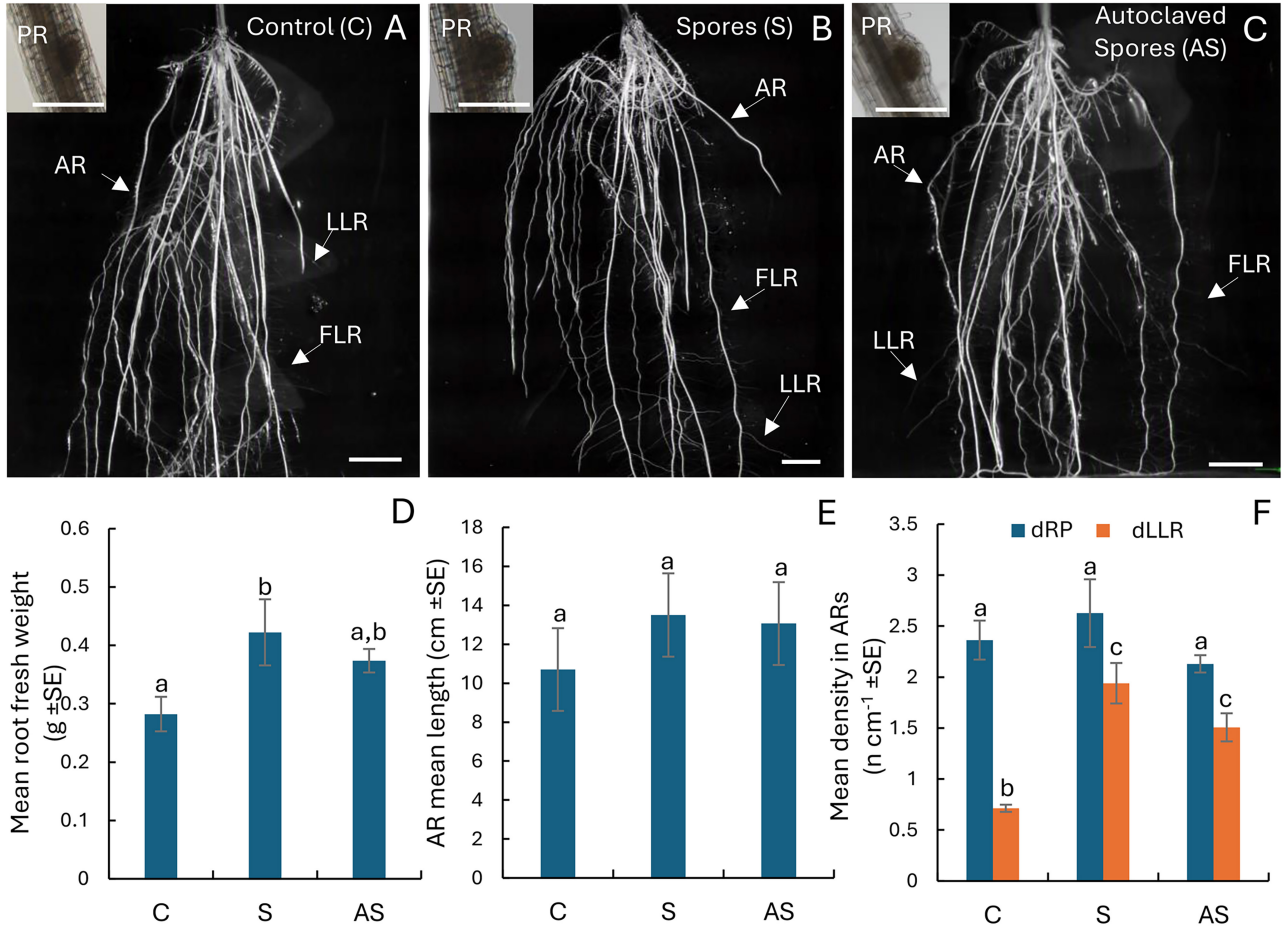
Root apparatus morphological analysis. (A) Root apparatus of the C group. (B) Root apparatus of the S group. (C) Root apparatus of the AS group. In A–C the respective root primordia (RP) in the upper left corner are shown (bar = 1000 μm); Adventitious roots (ARs) and lateral roots, large lateral roots (LLRs) and fine lateral roots (FLRs), are indicated in A, B, and C. The scalebar is 1 cm. (D) Mean root fresh weight in grams (g). (E) Mean adventitious root length in centimeters (cm). (F) Mean density of RP and LLRs in ARs (n cm^−1^). Significant differences among the groups within the same measurement are indicated with different letters. Columns sharing the same letter are not significantly different. Significant differences of at least *p* < 0.05 are shown (*N* = 30).

Preliminary experiments with varying spore numbers (100, 300, and 500 spores per sample) were carried out to select the spore number that elicited a clear morphological response in RSA (Figure S2). The 100‐spore group showed a more pronounced effect on root fresh weight (Figure S2A) and secondary primordia and lateral root densities in LLRs (Figure S2D) compared with the other groups; therefore, this spore number was selected for subsequent experiments. The root system architecture of non‐exposed (C) rice plants and plants exposed to 100 spores, either non‐autoclaved or autoclaved, is shown in Figure 1A–C. No significant differences were observed in the number of ARs per seedling among the groups. The morphological variations of the RSA were assessed by evaluating mean root fresh weight, adventitious root (AR) length, and the density of root primordia (RP) and large lateral roots (LLRs) in ARs (Figure 1D–F). Figure 1D, illustrates a significant increase (*p* < 0.01) in mean root fresh weight in the S‐treated group, compared with C, whereas the AS treatment did not differ significantly from the control. No significant differences were observed in shoot weight (Figure S3). Likewise, no significant differences were observed among groups in adventitious root length (Figure 1E). In the analysis of RP and LLR density in ARs, RP density did not show significant differences among the groups (Figure 1F). In contrast, the density of LLRs in both treated groups was significantly higher than that of the control. These results highlighted that the S group, in particular, had a stronger effect on LLR formation.

### 3.2 Intact Spores Promoted LLR Elongation and Increased Primordia and Lateral Root Formation

**FIGURE 2:**
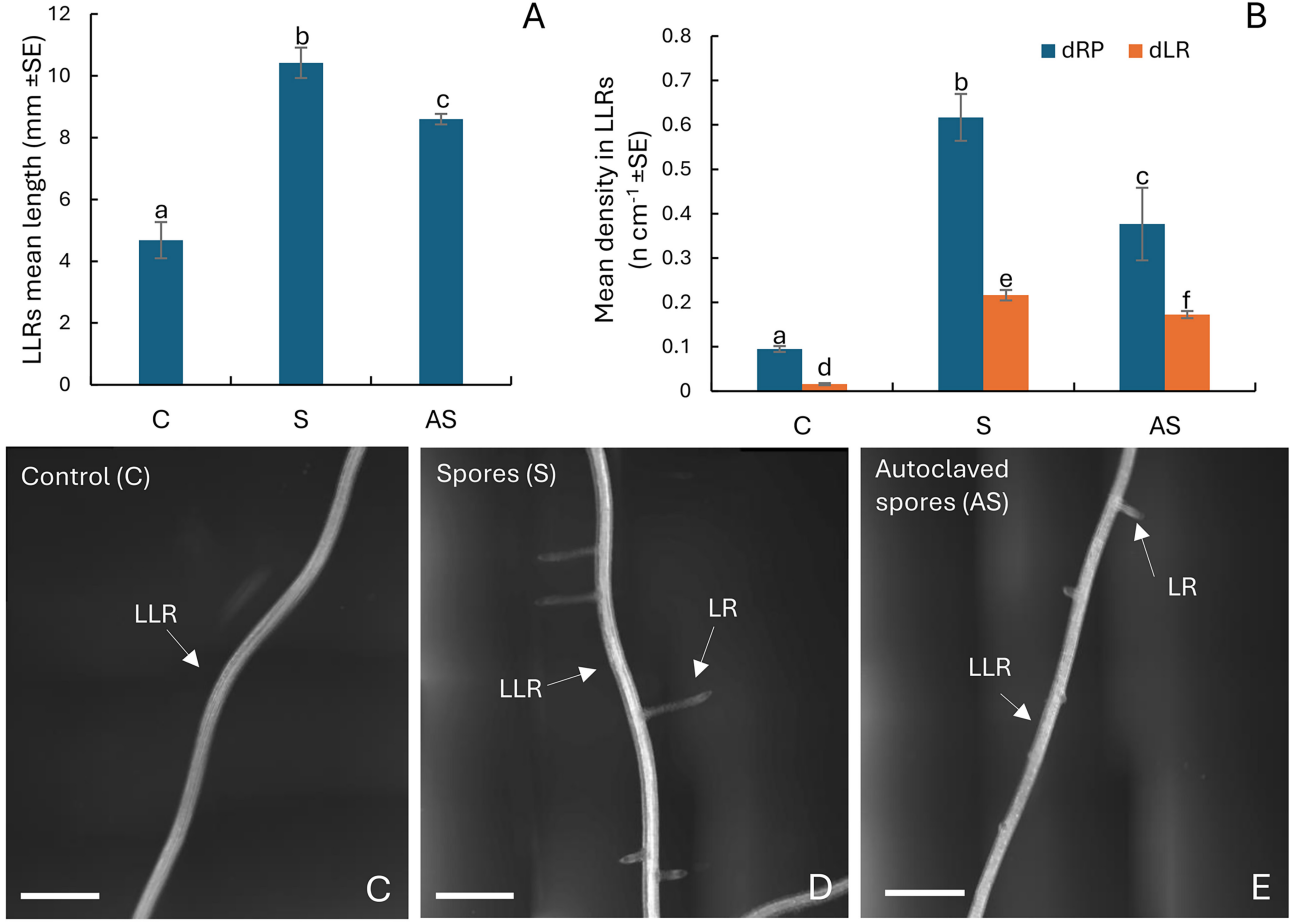
Large lateral roots (LLRs) morphological analysis. (A) Mean length of LLRs in millimeters (mm). (B) Mean density of root primordia (RP) and lateral roots (LR) in LLRs (n cm^−1^). (C) LLR of the C group, (D) LLR of the S group, (E) LLR of the AS group. The scalebar is 1 mm. Significant differences among groups within the same measurement are indicated with different letters. Significant differences of at least *p* < 0.05 are shown. (*N* = 30).

LLRs are preferential sites for arbuscular mycorrhizal (AM) symbiosis, particularly during the early stages of interaction in rice; therefore, their response to S and AS treatments provided key insights into plant‐AMF interactions. To investigate this, we analysed LLR development by quantifying the LLRs' length and density of RP and LRs present in LLRs (Figure 2). The mean LLRs' length in the S group was statistically greater than in all other groups (*p* < 0.01; Figure 2A). Furthermore, both RP and LR densities in LLRs were significantly higher in the S group compared with the AS and C groups (*p* < 0.01; Figure 2B). Consistently, Figure 2D showed a higher density of lateral roots in the S group relative to the AS group (Figure 2E), whereas the C group exhibited no visible lateral roots (Figure 2C).

Given that in rice endodermal cells cooperate with pericycle cells during lateral root primordium formation (Rebouillat et al. 2009), we further assessed whether root primordium formation was linked to differences in endodermal cell elongation in LLRs. The mean endodermal cell length was significantly higher in the S group (65.1 ± 1.07 μm) than in both the AS group (59.14 ± 0.29 μm, *p* < 0.05) and the C group (56.02 ± 2.41 μm, *p* < 0.01). These results indicated that the S treatment promoted LLR development by enhancing both elongation and the initiation and progression of RP and LR formation, likely in association with increased endodermal cell length.

### 3.3 Spore Perception Triggered Differential Auxin Accumulation and Systematic Depletion of Tissue Strigolactones

**FIGURE 3:**
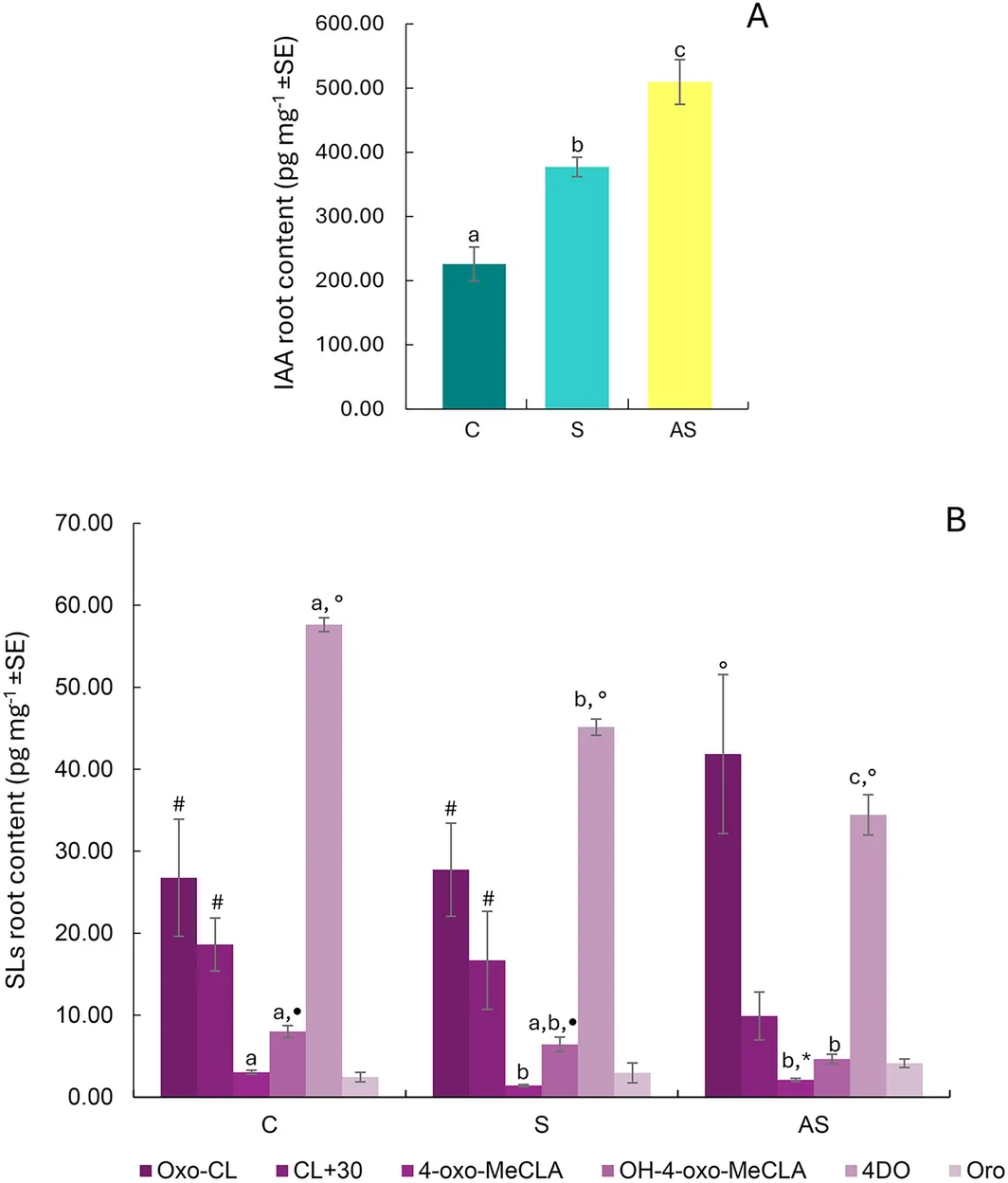
Auxin and Strigolactones content in the root apparatus. (A) Auxin (IAA) content in pg. mg^−1^ of dry weight. (B) Strigolactones (SLs) content (Oxo‐CL, CL + 30, 4‐oxo‐MeCLA, OH‐4‐oxo‐MeCLA, 4DO, Oro) in pg. mg^−1^ of dry weight. Significant differences are indicated with different letters among the groups for each hormone. Different symbols represent significant differences within the same the group. Columns without letters or symbols, or with the same ones, are not significantly different. Significant differences of at least *p* < 0.05 are shown. Means of three biological replicates.

The involvement of IAA and SLs during the pre‐symbiotic phase of the AMF–rice interaction was evaluated by quantifying their content in dry root biomass of the C, S, and AS groups. Figure 3A, shows significantly higher IAA levels in the S and AS groups compared with the C group, with significant differences also observed between the two exposed groups (*p* < 0.01). IAA content was also measured in shoots (Figure S4), and significantly higher IAA content was observed in the S group compared with the C and AS groups (*p* < 0.01).

Strigolactone (SL) content differed among the groups across both canonical (4‐deoxyorobanchol, 4DO; orobanchol, Oro) and non‐canonical compounds (Oxo‐CL; 4‐oxo‐19‐OH‐CL (CL+30); 4‐oxo‐MeCLA; and OH‐4‐oxo‐MeCLA; Figure 3B; Wang, Balakrishna, Martínez, et al. 2024). When considered along the biosynthetic pathway, distinct patterns emerged. Within the canonical SLs, 4DO levels were significantly reduced in both S and AS compared with C (*p* < 0.01), whereas Oro concentrations did not differ significantly among groups. Among non‐canonical SLs, Oxo‐CL accumulated to higher levels in the AS group relative to both C and S, whereas 4‐oxo‐19‐OH‐CL showed the opposite trend, with reduced levels in AS compared with C and S. The downstream metabolite 4‐oxo‐MeCLA was significantly decreased in both S and AS relative to C (*p* < 0.01), whereas OH‐4‐oxo‐MeCLA was specifically reduced in the AS group (*p* < 0.01) compared with both C and S. Overall, these results indicated a general decline in root SL levels in response to spore exposure, particularly under the AS condition, with differential modulation of specific intermediates along the pathway.

### 3.4 Spores Induced Tissue‐Specific Nitric Oxide Accumulation and Internal Relocalization in LLR Apexes

**FIGURE 4:**
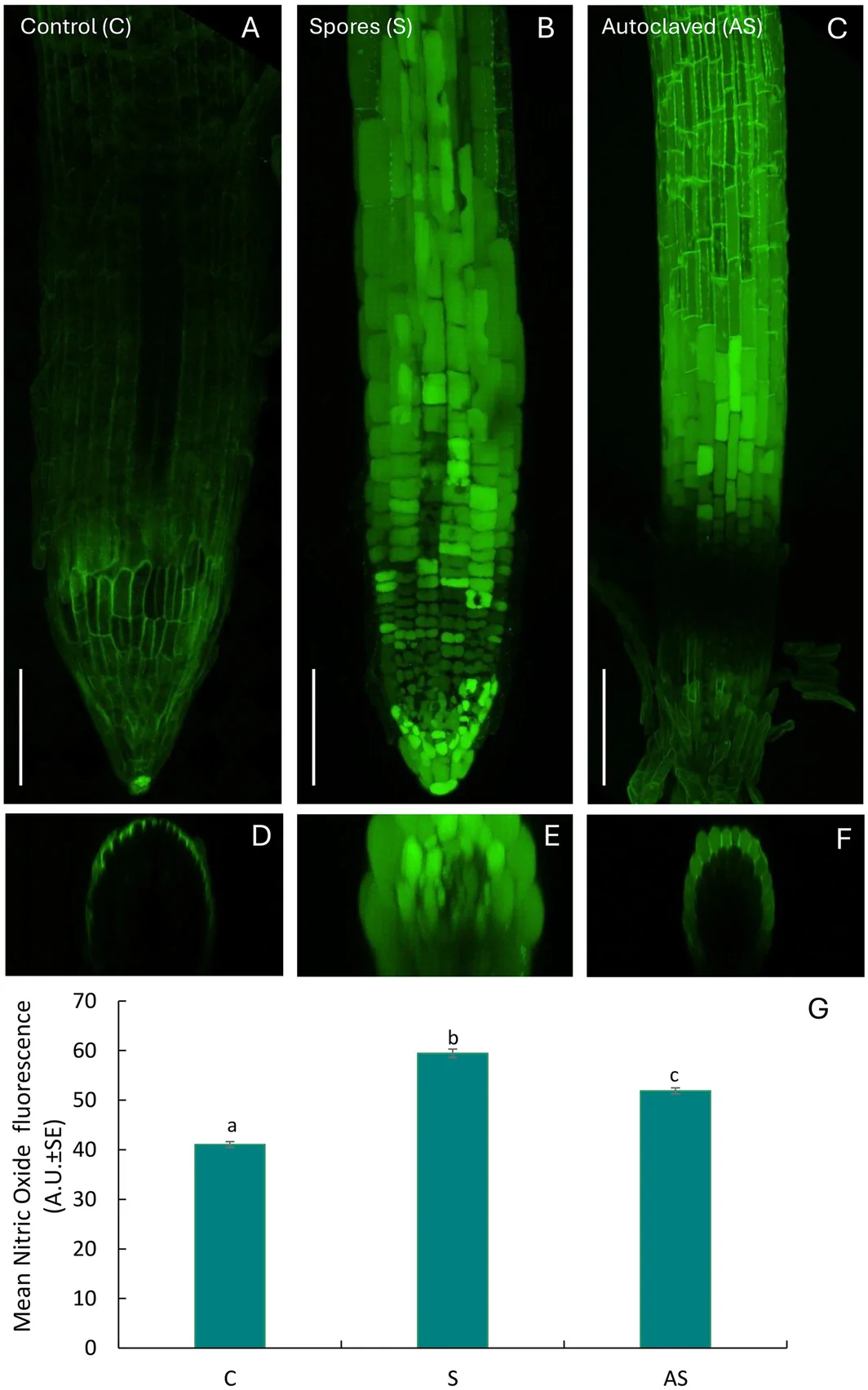
Nitric oxide (NO) histochemical detection. (A) A merged z‐stack image of the NO signal in a LLR from the C group. (B) A merged z‐stack image of the NO signal in a LLR from the S group. (C) A merged z‐stack image of the NO signal in LLRs from the AS group. The scalebar is 100 μm. (D) Transverse section of the z‐stack image of the LLR from the C group. (E) Transverse section of the z‐stack image of the LLR from the S group. (F) Transverse section of the z‐stack image of the LLR from the AS group. (G) Fluorescence intensity in the LLRs apex (apical region). Significant differences are indicated with different letters. Significant differences of at least *p* < 0.05 are shown (*N* = 30).

To assess the involvement of NO in response to spore presence, we analysed the presence and distribution of the NO signal in adventitious and large lateral roots. No NO signals were detected in adventitious roots; interestingly, an NO signal was observed in the apex region of the LLRs, where fluorescence intensity was measured (Figure 4G). The distribution of the NO signal in LLRs apexes is shown in Figure 4G. Regarding fluorescence intensity, measurements of the LLR apexes revealed significantly higher values in the S group compared with the C (*p* < 0.01) and AS groups (*p* < 0.05). To further evaluate NO signal distribution, representative roots from each group were selected and z‐stacked images of the NO signal were acquired and combined into a single reconstruction (Figure 4A–C). Interestingly, a clear difference in the internal distribution of the NO signal was visible in the individual z‐planes and in the sliced images obtained for each large lateral root (Figure 4D–F). The images clearly showed that an NO signal was present in the internal tissue layers of the S group, whereas in the C and AS groups it was detected only in the epidermis. In time‐lapse (Movie S1) and z‐stack acquisitions (Movie S2) of the elongation region of an S‐treated LLR, fluorescent particles were observed moving within epidermal cells of the large lateral roots. In cortical, endodermal and pericycle cells, the fluorescent probe signal revealed the presence of NO in the cell vacuole. Finally, the presence of an NO signal in the LLR samples was validated using the NO scavenger cPTIO (Figure S5), which removed the NO signal in the respective groups.

### 3.5 Transcriptomic Profiling Revealed Divergence Between Symbiotic Signaling and Stress‐Related Defense Responses

To characterize the transcriptional profile of the different groups, RNA sequencing was performed on LLR samples. At the transcriptome‐wide level, we first assessed the total number of expressed transcripts across groups. The Venn diagram (Figure S6) illustrates the distribution of these transcripts, highlighting the impact of AMF presence on the total gene repertoire. Specifically, 299 genes were detected exclusively in the C group, 582 in the S group, and 807 in the AS group, whereas 20 502 genes were common to all three groups.

Tables 1 and 2 list selected differentially expressed genes in the S and AS groups, respectively, compared with the control. In the S group (Table 1), genes related to IAA signaling were notably affected. For example, *OsIAA20*, a transcriptional repressor of IAA signaling, was downregulated, whereas *OsARF3*, an IAA response factor capable of activating or repressing gene expression in response to IAA, was upregulated. Additionally, *OsNRT2.1*, a high‐affinity nitrate transporter (Zhang et al. 2024), was upregulated, suggesting a possible role in the NO accumulation observed in LLRs. Furthermore, genes encoding Pathogenesis‐Related Protein 4 (*OsPR4c* and *OsPR4b*), typically expressed during pathogen attack but also responsive to beneficial microbes such as AMF, were activated, together with *OsPR1*, *PR1A*, *OsPR1#072*, and *OsPR1#073*.

**TABLE 1:** List of genes expressed in the LLRs of the S group compared to the C group.

Gene ID	Gene name	Gene regulation	Gene family	Description
Os06g0166500	OsIAA20	Downregulated	ARF	Auxin signal trasduction
Os01g0753500	OsARF3	Upregulated	ARF	Auxin signal trasduction
Os02g0112600	NRT2.1	Upregulated	CKX	Nitrogen metabolism
Os01g0940000	CKX	Upregulated	NRT2	Regulation of hormone levels
Os11g0592000	OsPR4c	Upregulated	PR4	Multi‐organism process
Os11g0592100	OsPR4b	Upregulated	PR1	Multi‐organism process
Os07g0129300	OsPR1	Upregulated	PR1	Plant hormone signal trasduction
Os07g0129200	PR1A	Upregulated	PR1	Plant hormone signal trasduction
Os07g0127500	OsPR1#072	Upregulated	PR1	Plant hormone signal trasduction
Os07g0127600	OsPR1#073	Upregulated	PR1	Plant hormone signal trasduction

*Note:* The table describes, starting from the left, the gene ID, the gene name, whether the gene is upregulated or downregulated, the name of the transcription factor family, and a brief description of their role. Mean of three biological replicates.

**TABLE 2:** List of genes expressed in the LLRs of the AS group compared to the C group.

Gene ID	Gene name	Gene regulation	Transcription factor family	Description
Os02g0769100	OsSAUR12	Upregulated	SAUR	Auxin signal trasduction
Os05g0143800	OsGH3‐6	Upregulated	GH3	Auxin signal trasduction
Os05g0186900	OsIAA16	Upregulated	ARF	Auxin signal trasduction
Os07g0127500	OsPR1#072	Upregulated	PR1	Multi‐organism process
Os07g0127600	OsPR1#073	Upregulated	PR1	Multi‐organism process
Os07g0129200	PR1A	Upregulated	PR1	Multi‐organism process
Os07g0129300	OsPR1	Upregulated	PR1	Multi‐organism process
Os03g0860100	PR1b	Upregulated	PR1	Multi‐organism process
Os10g0191300	OsPR1#101	Upregulated	PR1	Multi‐organism process
Os07g0687700	OsbZIP63	Upregulated	bZIP	Plant hormone signal trasduction
Os11g0152700	OsbZIP79	Upregulated	bZIP	Plant hormone signal trasduction
Os09g0280500	OsbZIP70	Upregulated	bZIP	Plant hormone signal trasduction
Os02g0194900	OsbZIP17	Upregulated	bZIP	Plant hormone signal trasduction
Os12g0152900	OsbZIP83	Upregulated	bZIP	Plant hormone signal trasduction
Os06g0614100	OsbZIP49	Upregulated	bZIP	Plant hormone signal trasduction
Os01g0867300	OsTGAP1	Upregulated	bZIP	Regulator of phytoalexin biosynthesis
Os07g0127700	BZIP12	Upregulated	bZIP	Plant hormone signal trasduction
Os10g0571300	OsJAZ11	Downregulated	JAZ	Jasmonate signal trasduction
Os04g0618700	ACS2	Upregulated	ACS	Ethylene biosynthesis
Os03g0860100	OsERF#083	Upregulated	ERF	Ethylene signal trasduction
Os04g0578000	Cht3	Upregulated	GH18	Defense‐related chitinase gene
Os07g0127700	Cht2	Upregulated	GH18	Defense‐related chitinase gene
Os06g0726200	Cht1(Chi1)	Upregulated	GH18	Defense‐related chitinase gene

*Note:* The table describes, starting from the left, the gene ID, the gene name, whether the gene is upregulated or downregulated, the name of the transcription factor family, and a brief description of their role. Mean of three biological replicates.

In the AS group (Table 2), several IAA‐related genes were upregulated, including *OsIAA16*, *OsGH3‐6*, and *OsSAUR12*. *OsIAA16* is a negative regulator of IAA signaling, *OsGH3‐6* conjugates excess IAA to amino acids to reduce free IAA levels, and *OsSAUR12* is an early IAA responsive gene rapidly induced by IAA. Genes from the *PR1* family (e.g., *OsPR1#072*, *OsPR1#073*, *OsPR1#101*, *OsPR1*, *PR1A*, *PR1b*) were also upregulated, consistent with the S group. Furthermore, several bZIP transcription factors associated with stress responses were induced (*OsbZIP63*, *OsbZIP79*, *OsbZIP70*, *OsbZIP17*, *OsbZIP83*, *OsbZIP49*, *BZIP12*), together with *OsTGAP1*, a key regulator of phytoalexin biosynthesis activated by jasmonic acid (JA). Defense‐related chitinase genes (*Cht1*, *Cht2*, *Cht3*) which are strongly induced by JA and ethylene, as well as *ACS2* (a gene involved in ethylene biosynthesis) and the ethylene responsive transcription factor *OsERF*#083 were also upregulated. In contrast, *OsJAZ11*, a repressor of the JA signaling pathway, was downregulated. The transcriptomic analyses revealed no differential expression of genes involved in SLs biosynthesis and signaling in the S and AS groups compared with the C group.

## 4 Discussion

The results presented in this study provide comprehensive insights into the effects of autoclaved and non‐autoclaved spores of the fungus *Rhizophagus irregularis* on root system architecture, phytohormones, and NO in *Oryza sativa* L. Morphological assessments, phytohormone detection, and NO histochemical analyses revealed significant changes in root structure, phytohormone modulation, and NO production, indicating that spore presence and viability play crucial roles in root development through IAA and SL modulation and stimulation of NO production.

Compared with the root system architecture of the control group, S‐ and AS‐treated groups showed distinct differences, particularly in the length and density of large lateral roots and in root fresh weight. Adventitious root length remained unchanged across all groups, indicating that spores did not affect AR elongation. Likewise, the density of adventitious root primordia remained unchanged, in contrast to previous reports (Chiu et al. 2022), which may be due to differences in experimental conditions and to the embryonic nature of roots at this stage, which is characterized by programmed development (Rebouillat et al. 2009; Betti et al. 2021). By contrast, the density of LLRs was significantly increased in both the S and AS groups compared with the control. This suggests that, in adventitious roots, S and AS treatment promoted LLR development at early stages, with induction likely mediated by chitin‐derived compounds released from the spore cell wall (Chiu et al. 2022; Raffaele et al. 2024).

In contrast, within the LLRs, which are preferred sites of symbiosis in rice (Gutjahr et al. 2009), elongation as well as the density of root primordia and lateral roots were significantly enhanced in the S group compared with the C and AS groups. These differences were further supported by measurements of endodermal cell length, which was significantly greater in the S group, indicating enhanced cellular elongation underlying LLR growth. The reduced elongation observed in the AS group relative to S suggests differential regulation of root growth, likely associated with hormonal imbalance rather than with simple absence of stimulatory signals. Indeed, quantification of indole‐3‐acetic acid (IAA) revealed elevated levels in both S and, more prominently, AS‐treated roots compared with controls. Although auxin is well established as a key promoter of lateral root initiation and primordia formation in rice (Nakamura et al. 2023), its effect on root elongation is dose‐dependent, with supra‐optimal concentrations inhibiting cell elongation (Ivanchenko et al. 2010). In this context, the lower LLR elongation observed in the AS group may be interpreted as a consequence of auxin overaccumulation or dysregulated auxin homeostasis. This interpretation is supported by transcriptomic data showing upregulation of auxin‐responsive repressors such as *OsIAA16* and the conjugating enzyme *OsGH3‐6* in AS‐treated roots, both of which are typically induced under high‐auxin conditions to buffer excess hormone levels (Chen et al. 2022). Such a response suggests activation of a negative feedback mechanism aimed at mitigating auxin toxicity. Conversely, in the S group, upregulation of *OsARF3* and downregulation of the repressor *OsIAA20* indicate a more balanced and transcriptionally active auxin signaling state, consistent with conditions favoring both primordia formation and sustained root elongation. Moreover, *OsNRT2.1* has been linked to auxin signaling, as its overexpression induces the expression of auxin transporters (OsPINs), thereby promoting root system development, including lateral root formation in rice (Naz et al. 2019). This suggests that, in the S group, auxin distribution and transport may have been more efficiently coordinated, further supporting optimal LLR development. Together, these findings support a model in which S promoted optimal auxin signaling conducive to LLR development, whereas AS induced an excessive or misregulated auxin response that enhanced initiation processes but restricted elongation through inhibition of cell expansion.

Concerning SL dynamics within root tissues, our results revealed a general decline in both canonical and non‐canonical SLs following spore exposure. The reduction in 4‐deoxyorobanchol (4DO), one of the canonical SLs known to stimulate hyphal branching in AM fungi, is consistent with feedback regulation of SL biosynthesis upon fungal perception. Such regulation has been proposed to fine‐tune SL availability during the transition from the pre‐symbiotic to the early symbiotic phase (Fiorilli et al. 2019), although the relative contribution of internal versus exuded SL pools remains to be clarified (Wang, Chen, Braguy, and Al‐Babili 2024). In addition, SLs are known to interact with auxin pathways, primarily by modulating auxin transport and distribution rather than directly controlling auxin biosynthesis (Hayward et al. 2009; Prusinkiewicz et al. 2009). Therefore, the observed decline in 4DO has contributed to shifts in auxin homeostasis, potentially contributing to the prevention of excessive auxin accumulation (Chen et al. 2022). A similar trend was observed for noncanonical SLs, including 4‐oxo‐MeCLA and OH‐4‐oxo‐MeCLA. Although their specific biological roles remain less well characterized than those of canonical SLs, these compounds are thought to participate in SL‐mediated developmental regulation (Li, Chen, et al. 2024). In rice, SLs have been reported to suppress lateral root development, largely through interactions with auxin signaling (Sun et al. 2019). Accordingly, the reduction in SL levels observed in the S group may reflect a partial release of this suppression, thereby facilitating lateral root elongation. In contrast, the pronounced depletion of SL metabolites in the AS group, together with elevated IAA levels, may reflect a disruption of hormonal balance, potentially leading to altered or dysregulated root development rather than coordinated growth promotion.

Interestingly, RNA‐seq analysis revealed no significant differential expression of SL biosynthetic or signaling genes at the sampled time point. This suggests that the observed metabolic changes in SL levels have been regulated post‐transcriptionally or may have occurred during a transcriptional window outside our analysis. However, the transcriptomic profile of the AS group was characterized by robust upregulation of stress and defense related genes (e.g., *PR* genes, *bZIP* genes, etc.). This implies that the plant did not recognize the spores as symbionts, but rather as a source of biotic stress. This stress‐induced state could further suppress SL production while maintaining high IAA signaling as part of a generalized defense response (Pandey et al. 2016; Bouzroud et al. 2018), ultimately restricting the optimal development of LLRs observed in the S group.

Histochemical analysis revealed that the S group stimulated NO production within the internal tissues of the LLR apex. In contrast, LLRs of the AS and C groups exhibited only superficial NO signaling localized in the epidermal tissues. Quantification of NO fluorescence intensity (Figure 4G) further highlighted significant differences between the S and AS groups, suggesting that chitin‐derived compounds or other molecules released by autoclaved spores were insufficient to induce NO production at levels comparable to those triggered by live spores. Transcriptomic analysis supported these findings, with upregulation of *NRT2.1* in S‐exposed LLRs, a high affinity nitrate transporter (Wang, Balakrishna, Martínez, et al. 2024). Since nitrate is one of the putative precursors of NO (Reda et al. 2022), its increase may have contributed to enhance NO production. Therefore, these results further support a dynamic relationship between IAA and NO at the expression level during spore‐induced root responses. Interestingly, a slight signal was also observed in the apical and elongation zones of LLRs in the AS group compared with the C groups (Figure 4C). This indicates that even autoclaved spores partially activated NO signaling in regions of active root growth, consistent with the induction of LLRs observed in the morphological analysis. A nitric oxide signal was detected in the control group predominantly in the outermost tissues and root cap, supporting its constitutive role in root development (Prakash et al. 2020; Das et al. 2026). In contrast, spore exposure altered the spatial distribution of NO within the root, suggesting a role for biotic cues in shaping NO‐mediated signaling patterns. In the S‐treated roots, vesicle‐like particles observed especially in the rhizodermal cells (Movies S1 and S2), suggested vesicle trafficking potentially linked to cell wall construction and possibly associated with NO containing vesicles (Lombardo and Lamattina 2012), although further testing is needed to confirm this hypothesis. This observation aligns with previous reports showing that NO mediates IAA responses and cell wall biosynthesis during lateral root formation (Correa‐Aragunde et al. 2008); however more studies are required to validate the observations reported here.

In conclusion, our results demonstrated a differential response of rice roots to non‐autoclaved versus autoclaved AMF spores, particularly in LLRs. In the S group, LLR elongation and secondary branching were significantly promoted, accompanied by elevated IAA levels, reduced SLs, and upregulation of IAA‐responsive genes. In contrast, autoclaved spores reduced LLR elongation and branching and triggered a distinct signaling profile characterized by the induction of IAA repressors and binding proteins together with stress‐ and defense‐related genes. NO production and localization mirrored these differences, with substantially higher NO accumulation in roots exposed to non‐autoclaved spores. These findings underscore the dynamic and multifaceted responses of rice roots to AMF‐derived signals and highlight the crucial influence of root type in determining these responses, beyond the effects of either non‐autoclaved spores or chitin‐derived compounds released from autoclaved spores.

Collectively, this study revealed a complex modulation of IAA, SLs, and NO in regulating root architecture during the pre‐symbiotic phase of AMF–plant interactions. Elucidating these early signaling events is of critical importance, as they not only act as a discrimination mechanism between a beneficial symbiont and a potential pathogen, but also drive the hormonal shifts required to optimize root development for successful symbiosis establishment. Further research is needed to unravel the exact molecular pathways orchestrating these early events and their long‐term role in establishing successful symbiosis.

## Author Contributions

G.R. performed the experiments, analysed the data, and wrote the manuscript. M.R. contributed to the experimental design, data analysis, and manuscript writing. E.D.D. contributed to data analysis and video acquisition. J.Y.W. carried out the hormonal analyses. P.B. assisted with NO z‐stack image acquisition. C.F. supported the microscopic image analysis. S.A. supported the hormonal analyses. L.D.G. revised the manuscript. B.M. directed the research, secured funding, and revised the manuscript. All authors read and approved the final version of the manuscript.

## Funding

This work has received funding from the European Research Council (ERC) under the European Union's Horizon 2020 Research and Innovation Programme grant agreement no. 101003304 (I‐Wood).

## Disclosure

No generative AI tools were used in preparing this manuscript.

## Conflicts of Interest

The authors declare no conflicts of interest.

## Supporting information


**Movie S1:** Time‐lapse of NO in large lateral roots exposed to spores.

**Movie S2:** Z‐stack image of NO in large lateral roots exposed to spores.

**Figure S1:** Autoclaved spores stained with NBT and acid fuchsin.
**Figure S2:** Preliminary study with different spore exposure number.
**Figure S3:** Mean shoots fresh weight expressed in grams.
**Figure S4:** Auxin content in shoots expressed in pg. mg^−1^.
**Figure S5:** NO fluorescence in the presence of cPTIO scavenger.
**Figure S6:** Venn diagram of detected transcripts across C, S, and AS groups.

## Data Availability

All data needed to support the conclusions of this manuscript are included in the main text and Supporting Information.
