# Supplementary material for: Auxin, Strigolactones, and Nitric Oxide Coordinate Lateral Root Development in Rice During Early Interaction With Rhizophagus irregularis
Source: Physiol Plant. 2026 Sep 8;178(5):e71099. doi: 10.1111/ppl.71099 (PMC13553156; doi:10.1111/ppl.71099)
Supplement: Supplementary file 1 [file PPL-178-e71099-s003.docx]

Supporting Information

Auxin, Strigolactones, and Nitric Oxide Coordinate Lateral Root Development in Rice During Early Interaction with *Rhizophagus irregularis*

*Giulia Raffaele^1,2,3*,a^, Marilena Ronzan^1*,a^, Emanuela Del Dottore^1^, Jian You Wang^5,6^, Paolo Bianchini^4^, Alberto Diaspro^4,8^, Carlo Filippeschi^1^, Salim Al-Babili^5^, Laura De Gara^2^; Barbara Mazzolai^1^**

*Giulia Raffaele, Marilena Ronzan, Barbara Mazzolai **emails:** giulia.raffaele@unina.it**,** marilena.ronzan@iit.it, barbara.mazzolai@iit.it

^a^ These authors equally contributed to the work

**This PDF file includes:**

**-Figure S1.** Autoclaved spores stained with NBT and acid fuchsin.

**-Figure S2.** Preliminary study with different spore exposure number.

**-Figure S3.** Mean shoots fresh weight expressed in grams.

**-Figure S4.** Auxin content in shoots expressed in pg mg^-1^.

**-Figure S5.** NO fluorescence in the presence of cPTIO scavenger.

**-Figure S6.** Venn diagram of detected transcripts across C, S, and AS groups.

**Other Supplementary Materials for this manuscript include the following:**

-**Movie S1**. Time-lapse of NO in large lateral roots exposed to spores.

-**Movie S2.** Z-stack image of NO in large lateral roots exposed to spores.

**Figure S1.** Autoclaved spores stained with NBT and acid fuchsin. Hyphae and autoclaved spores of the AM fungus *R. irregularis* after dual staining with NBT and acid fuchsin (pink contrast solution). The colourless, transparent, or faint yellowish, appearance of the autoclaved spores indicate the absence of enzymatic activity, which would otherwise reduce NBT to formazan and form a dark blue precipitate inside the spores. Scale bar: 100 µm.

**Figure S2.** Preliminary study with different spore exposure number. The treatments indicated consist of a C group (control), T100 group (100 spores treatment), T300 group (300 spores treatment) and T500 group (500 spores treatment). (A) Mean root fresh weight in grams (g); (B) Adventitious roots mean length in centimetres (cm); (C) Mean number of lateral roots and primordia in LLRs. (D) Density of lateral roots and primordia in LLRs. Significant differences are indicated with different letters. Columns sharing the same letter are not significantly different. Significant differences of at least P< 0.05 are shown (N = 20).

**Figure S3.** Mean shoot fresh weight expressed in grams (g). Columns sharing the same letter are not significantly different (N = 30).

**Figure S4.** Auxin content in shoots expressed in pg mg^-1^. Significant differences are indicated with different letters. Columns sharing the same letter are not significantly different. Significant differences of at least P< 0.05 are shown. Means of 3 biological replicates.

**Figure S5.** NO fluorescence in the presence of cPTIO scavenger. (A) cPTIO effect on a LLR from the C group; (B) cPTIO effect on a LLR from the S group (C) cPTIO effect on a LLR from the AS group. The scalebar is 100 µm.

**Figure S6.** Venn diagram of detected transcripts across C, S, and AS groups. The diagram displays the number of unique and common genes detected in each group, representing the total gene repertoire under the influence of AM fungal presence. A total of 20,502 genes were expressed in common across all groups; 299, 582, and 807 genes were exclusively detected in the C, S, and AS groups, respectively. Values represent the mean of 3 biological replicates.
